# Accuracy assessment of dental age estimation with the Willems, Demirjian and Nolla methods in Spanish children: Comparative cross-sectional study

**DOI:** 10.1186/s12887-020-02247-x

**Published:** 2020-07-31

**Authors:** Marta Macarena Paz Cortés, Rosa Rojo, Esther Alía García, Maria Rosa Mourelle Martínez

**Affiliations:** 1 Faculty of Dentistry, Alfonso X El Sabio University, Villanueva de la Cañada, 28691 Madrid, Spain; 2 Department of Dental Clinical Specialties, School of Dentistry, Complutense University, 28040 Madrid, Spain

**Keywords:** Dental age estimation, Nolla method, Willems method, Demirjian method, forensic odontology, Spanish

## Abstract

**Background:**

The objective of this study was to evaluate and compare the validity and accuracy of the Willems, Demirjian and Nolla methods in predicting chronological age in a Spanish ethnicity population.

**Methods:**

A sample of 604 orthopantomographs of Spanish children aged 4 to 13 years was evaluated by two independent evaluators. Descriptive statistics were applied to calculate the chronological age and dental age, presenting the mean and standard deviation. The difference between dental age and chronological age was calculated for each method. A positive result indicated an overestimation and a negative figure indicated an underestimation. The Wilcoxon test for paired data and Spearman’s correlation coefficient were applied by age groups and sex to compare the chronological age and dental age of each method (that of Willems, Demirjian and Nolla). Statistical tests were performed at a 95% confidence level.

**Results:**

The interexaminer agreement was 0.98 (p = 0.00), and the intraexaminer agreement was 0.99 (p = 0.00). The Willems method significantly overestimated the age of boys (0.35 years (0.93)) and girls (0.17 years (0.88)). The Demirjian method significantly overestimated the age of boys (0.68 years (0.95)) and girls (0.73 years (0.94)). The Nolla method significantly underestimated age in boys (0.44 years (0.93)) and girls (0.82 years (0.98)).

**Conclusions:**

In the Spanish population, the use of the Demirjian method for legal and medical purposes is frequent. This study reveals that the Willems method is more appropriate due to its greater precision in estimating dental age.

## Background

The estimation of chronological age is used as a clinical tool in the field of pediatric dentistry and orthodontics, allowing us to assess the progress or the most appropriate treatment of different dental malocclusions based on craniofacial growth in children [1, 2]. It also has great value in legal and anthropological medical studies [1], providing information on past populations and helping in the identification of deceased persons or in immigration matters, clarifying the age of living people whose data are doubtful or nonexistent [2].

There is no consensus on what is the best method to predict chronological age [3]. Bone growth evaluation has been used on wrist radiographs [4, 5] or according to the stages of maturation of the cervical vertebrae [6–8]. Currently, one of the most commonly used methods to estimate chronological age is the calculation of dental age through the mineralization phases of the teeth. This method is accepted and recognized because it shows little variation compared to other properties related to skeletal or sexual growth. However, hereditary, functional, environmental, sex, nutritional and metabolic factors must be taken into account since the specific standards of each population are important for the application of these methods. Therefore, assessing the accuracy and applicability of age estimation methods for different populations is of vital importance [3].

The Nolla method is used in clinical practice and teaching within the field of dentistry. Although it presents a high degree of intra-observer agreement (greater than 90%) [9], this method has been less used and tested in different populations [10], and only one occasion in the Spanish population [11].

The Demirjian method [12, 13] is one of the most popular tools for predicting chronological age due to its simplicity, the degree of intraexaminer agreement and the ease of its standardization and reproducibility [3]. In the Spanish population, the Demirjian method is recommended by the Institutes of Legal Medicine of Spain [14] for legal and medical purposes, and it has been used in numerous studies independently [6, 15–18] or together with the Nolla method [1, 10, 19].

The Willems method [20] was a modification of the Demirjian method [12] published in 1973. It has been applied to different populations, observing that it provides comparatively smaller overestimations than other methods [21] and that the estimate is even more precise [22] in some populations. However, the Willems method cannot be used as a global tool due to the differences between ethnicities [23]. In Spanish ethnicity, it has been applied only to a sample of 266 children compared to the Demirjian and Cameriere methods [24].

The objective of the study was to evaluate and compare the validity and accuracy of the Willems, Demirjian and Nolla methods in predicting chronological age in Spanish ethnicity.

## Methods

The Strengthening the Reporting of Observational studies in Epidemiology (STROBE) [25] recommendation guidelines were followed in this study and was conducted in accordance with the Declaration of Helsinki [26]. A cross-sectional design was carried out using panoramic radiographs of 604 patients (302 boys and 302 girls) of Spanish ethnicity from five different clinics in the community of Madrid (Hortaleza, Carabanchel, Campamento, Arganzuela, and the city center). Patients were recruited between 2004 and 2015, and the data were analyzed between January and December 2017.

### Inclusion and exclusion criteria

The inclusion criteria were as follows: radiographs of children between 4 and 13 years of age, with images saved in JPEG (Joint Photographic Experts Group) format, whose sex, place and date of birth of the child, as well as the Spanish origin of parents and grandparents were included in the medical record. In order to compare the three methods (Willems, Demirjian, and Nolla), the radiographs with the presence of seven permanent mandibular teeth on the left side are evaluated.

The exclusion criteria were radiographs where the date of birth and sex were not registered; poor-quality radiographs that did not allow proper visualization of the degree of dental development; or radiographs from children with systematic diseases, syndromes or alterations in dental development, permanent tooth extraction (except for the third molar), the use of orthodontic appliances or a history of dental trauma.

### Obtaining and management of radiographs

Radiographs were selected using the random function of the Excel 14.0 (Microsoft Office, Redmond, Washington, USA) program in a previous list of potential children who met the inclusion criteria. All panoramic radiographs of the subjects were obtained with the same Orthodox 2D1 X-ray device model (Siemens, Spain) and were saved in JPEG format. The radiographs were analyzed by two independent evaluators (Marta Paz Cortes and Rosa Rojo) on two computers with AMD Ryzen 5-3500 U, 8 GB RAM, 1 TB HDD + 256 GB SSD, AMD Radeon Vega HD 7950 graphics cards with 27 GB 1920_1080 resolution LED monitors and Intel Core i7 processors. The evaluators were blinded concerning the chronological age of the patient. The following data were registered in a data collection notebook (CRF): clinic history number, dental clinic, the date of birth, the date of the X-ray, sex, and the degree of dental calcification according to the Demirjian and Nolla methods.

### Methodology for the calculation of age

#### Chronological age (CA)

The chronological age was calculated by subtracting the date of the radiography from the date of birth.

#### Dental age (DA)

 Dental age was calculated according to the degree of dental development using three methods: the Demirjian, Willems, and Nolla methods.

The CA was subtracted from the DA and a positive result indicates an overestimation and a negative figure an underestimation.

The Demirjian [27] method assesses the degree of development of each of the mandibular teeth on the left side (except the third molar) by classifying them on an 8-stage scale represented by the letters “A” through “H”. A score is assigned to each of the seven teeth according to their degree of mineralization. The stage represented by the letter is converted to a score, according to sex, using a conversion table developed by the authors. All the numerical scores are added, and the result is converted to dental age, according to sex, by referring to another table.

The Willems [20] method assesses the degree of development of each of the mandibular teeth on the left side (except the third molar) using the classification of the method proposed by Demirjian. A score is assigned to each of the seven teeth, which is converted to an average score, according to sex, in a calculation developed by the authors. All the values are added, and the result corresponds to the dental age.

The Nolla [28] method assesses the degree of dental development of the teeth of the mandibular and maxillary teeth on the left side (except the third molar) by classifying it into ten degrees of dental development. A score is assigned to each of the teeth, which is converted to an average score, according to sex, in a calculation developed by the authors. All the values are added, and the result corresponds to the dental age.

### Reproducibility of measurements

After the evaluation of 20 radiographs, the two evaluators had a rest period of 10 min (maximum analyzed 100 radiographs per day). After the evaluation of all radiographs and after 8 weeks, one of the evaluators (Marta Paz Cortes) reevaluated 100% of the radiographs of the total sample using the Willems, Demirjian and Nolla methods. Their selection of the order of radiographs was made randomly with the random Excel command.

The sample size calculation was based on a 95% confidence interval, a power of 80% and an effect size of 0.30, taking into account the data published in the study by Feijóo et al. [16] about the Spanish population with the Demirjian method. We used the difference of means between the real age and the chronological age in children (0.87 years) and the standard deviation (2.95 years) assuming the null hypothesis, in which there are no differences between the real age and the chronological age.

### Statistical analysis

Descriptive statistics were applied to calculate the chronological age and dental age, presenting the mean and standard deviation. The difference between DA and CA was calculated for each method. A positive result indicated an overestimation and negative figure of an underestimation. The Shapiro-Wilk test was applied to determine the normality of the data, which showed a nonparametric distribution. The Wilcoxon test for paired data was applied by age groups and sex to compare the chronological age and dental age of each method (Willems, Demirjian and Nolla). Spearman’s correlation coefficient was applied to assess the correlation between the chronological age and dental age of each method (Willems, Demirjian and Nolla). A linear regression model was used to obtain a parsimonious model allowing the chronological age to be estimated from the measurements taken of the seven mandibular left teeth with each of the methods and grouped by sex. Kappa statistics were used to assess inter- and intraobserver reliability for the Demirjian and Nolla methods by age group. The results of the agreement with the Willems method were not reported since the calculation that is made is based on the estimation data of the different stages of the maturation of the Demirjian method. To perform the sample calculation, the paired test was used to compare correlated measures specifying the standard deviations of the differences. Statistical tests were performed at a 95% confidence level with the Stata 11.1 software package (Stata Corp, College Station, TX, USA).

## Results

The mean chronological age of the entire sample was 8.77 years (1.94), of which that of boys and girls was 8.84 years (2.01) and 8.71 years (1.88), respectively. The distribution by age group of the total sample and according to sex is shown in Table 1.

**Table 1:** Distribution of the sample by age groups and sex

Age groups	Total	Mean	SD	Girls	Boys
4-6.9	122	6.43	0.52	59	63
7-7.9	119	7.55	0.30	65	54
8-8.9	136	8.45	0.29	69	67
9-10.9	130	9.93	0.57	67	63
11-13.9	97	12.14	0.61	42	55
Total	604			302	302
SD Standard deviation					

The global inter and intra examiner agreement for the Demirjian method was 0.980 and 0.991, respectively, and for the Nolla method 0.981 and 0.992, respectively. These results were statistically significant and showed an almost perfect agreement. The age range where the least degree of concordance was observed was between 7 and 7.9 years in both methods; however, the agreement was higher than 91% (Table 2). Data from the first examiner (Marta Paz Cortes) were used for data analysis.

**Table 2:** Results of the degree of inter and intra observer agreement. All results with *p* < 0.05

**Age groups**	Inter-examiner		Intra-examiner	
	**Demirjian**	**Nolla**	**Demirjian**	**Nolla**
4-6.9	0.94	0.94	0.94	0.95
7-7.9	0.91	0.92	0.92	0.93
8-8.9	0.92	0.93	0.93	0.94
9-10.9	0.95	0.97	0.99	0.99
11-13.9	0.98	0.99	0.99	0.99
Total	0.98	0.98	0.99	0.99

In this study, the mean dental age calculated with the Willems method was generally 9.04 years (1.99), of which that of boys and girls was 9.19 years (2.04) and 8.88 years (1.93), respectively. In the group of boys, the Willems method tends to overestimate, and although in the group of girls between 4 and 7.9 years and 9 to 0.9, this trend continues, there are age ranges that are prone to underestimation. In both groups, there is greater precision in estimating age between 11 and 13.9 years (Table 3).

**Table 3:** Results of the calculation of the dental age with the Willems method. The Wilcoxon test for paired data was applied by age groups and sex to compare the chronological age and dental age. Statistical tests were performed at a 95% confidence level (*p* ≤ 0.05). *DA * Dental age, *CA * Chronological age, *Diff.SD *Standard deviation differences, *SD *Standard deviation, *O *Overestimation, *U *Underestimation

**Age groups**	**n**	**CA**		**DA Willems**		***p****-value*	**CA-DA**	**Diff.SD**	**Trend**
		**mean**	**SD**	**mean**	**SD**				
**Girls**									
4-6.9	59	6.46	0.48	7.00	0.89	0.000	-0.54	0.70	O
7-7.9	65	7.50	0.30	7.77	0.79	0.008	-0.26	0.73	O
8-8.9	69	8.45	0.28	8.37	0.60	0.145	0.08	0.59	U
9-10.9	67	9.97	0.57	10.13	1.25	0.321	-0.16	1.05	O
11-13.9	42	12.14	0.63	12.08	1.32	0.965	0.05	1.22	U
Total	302								
**Boys**									
4-6.9	63	6.40	0.55	6.88	1.24	0.000	-0.48	0.93	O
7-7.9	54	7.60	0.28	8.26	1.09	0.000	-0.66	1.04	O
8-8.9	67	8.45	0.29	8.81	0.76	0.000	-0.36	0.67	O
9-10.9	63	9.89	0.58	10.11	1.16	0.163	-0.23	1.03	O
11-13.9	55	12.15	0.60	12.16	0.88	0.782	-0.01	0.87	O
Total	302								

The mean dental age calculated with the Demirjian method was in general 9.48 (2.08), of which that of boys and girls was 9.52 (2.11) and 9.44 (2.05), respectively. In both groups, the tendency was to overestimate. In boys, the Demirjian method was more accurate between 11 and 13.9 years and in girls between 8 and 8.9 years (Table 4).

**Table 4:** Results of the calculation of the dental age with the Demirjian method. The Wilcoxon test for paired data was applied by age groups and sex to compare the chronological age and dental age

**Age groups**	**n**	**CA**		**DA Demirjian**		***p****-value*	**CA-DA**	**Diff.SD**	**Trend**
		**mean**	**SD**	**mean**	**SD**				
**Girls**									
4-6.9	59	6.46	0.48	7.48	0.82	0.000	-1.02	0.65	O
7-7.9	65	7.50	0.30	8.20	0.83	0.000	-0.69	0.78	O
8-8.9	69	8.45	0.28	8.92	0.79	0.000	-0.48	0.76	O
9-10.9	67	9.97	0.57	10.77	1.34	0.000	-0.80	1.18	O
11-13.9	42	12.14	0.63	12.84	1.28	0.000	-0.70	1.21	O
Total	302								
**Boys**									
4-6.9	63	6.40	0.55	7.28	1.09	0.000	-0.88	0.78	O
7-7.9	54	7.60	0.28	8.43	1.04	0.000	-0.84	0.99	O
8-8.9	67	8.45	0.29	9.03	0.87	0.000	-0.57	0.76	O
9-10.9	63	9.89	0.58	10.47	1.31	0.001	-0.59	1.63	O
11-13.9	55	12.15	0.60	12.65	1.00	0.000	-0.50	1.00	O
Total	302								

The mean dental age calculated with the Nolla method was generally 8.14 years (1.82), of which that of boys and girls was 8.40 years (1.81) and 7.88 years (1.80), respectively. In both groups, the Nolla method tends to underestimate, except children between 4 and 6.9 years. This method was more accurate in the first age range for both boys and girls (Table 5).

**Table 5:** Results of the calculation of the dental age with the Nolla method. The Wilcoxon test for paired data was applied by age groups and sex to compare the chronological age and dental age

**Age groups**	**n**	**CA**		**DA Nolla**		***p****-value*	**CA-DA**	**Diff.SD**	**Trend**
		**mean**	**SD**	**mean**	**SD**				
**Girls**									
4-6.9	59	6.46	0.48	6.17	0.72	0.000	0.29	0.60	U
7-7.9	65	7.50	0.30	6.85	0.81	0.000	0.66	0.76	U
8-8.9	69	8.45	0.28	7.51	0.83	0.000	0.94	0.81	U
9-10.9	67	9.97	0.57	8.97	1.00	0.000	1.00	0.96	U
11-13.9	42	12.14	0.63	10.79	1.59	0.000	1.35	1.49	U
Total	302								
**Boys**									
4-6.9	63	6.40	0.55	6.43	0.91	0.813	-0.03	0.69	O
7-7.9	54	7.60	0.28	7.52	0.93	0.355	0.77	0.87	U
8-8.9	67	8.45	0.29	7.99	0.69	0.000	0.47	0.60	U
9-10.9	63	9.89	0.58	9.24	1.10	0.000	0.65	1.02	U
11-13.9	55	12.15	0.60	11.05	0.97	0.000	1.10	1.00	U

In general, the Willems and Dermirjian method significantly overestimate in both sexes. However, the Nolla method tended to underestimate. Among the three methods, the most accurate for estimating age in both sexes was the Willems method (Table 6).

**Table 6:** General and sex results of the comparison of chronological age with each dental method. The Wilcoxon test for paired data was applied

Method	n	CA		DA of the method		*p*-value	CA-DA	Diff.SD	Trend
		**mean**	**SD**	**mean**	**SD**				
Willems method for boys	302	8.71	1.88	8.88	1.93	0.001	-0.17	0.88	O
Willems method for girls	302	8.84	2.00	9.19	2.04	0.000	-0.35	0.93	O
Willems method	604	8.77	1.94	9.04	1.99	0.000	-0.26	0.91	O
Demirjian method for boys	302	8.71	1.88	9.44	2.05	0.000	-0.73	0.94	O
Demirjian method for girls	302	8.84	2.00	9.52	2.11	0.000	-0.68	0.95	O
Demirjian method	604	8.77	1.94	9.48	2.08	0.000	-0.70	0.95	O
Nolla method for boys	302	8.71	1.88	7.88	1.80	0.000	0.82	0.98	U
Nolla method for girls	302	8.84	2.00	8.40	1.81	0.000	0.44	0.93	U
Nolla method	604	8.77	1.94	8.14	1.82	0.000	0.63	0.97	U

Spearman’s correlation coefficients for girls and boys show strong linear correlations between chronological age and dental age for all methods; the rho values range from 0.86 to 0.89 and are significant in all cases (p = 0.00).

**Fig. 1:**
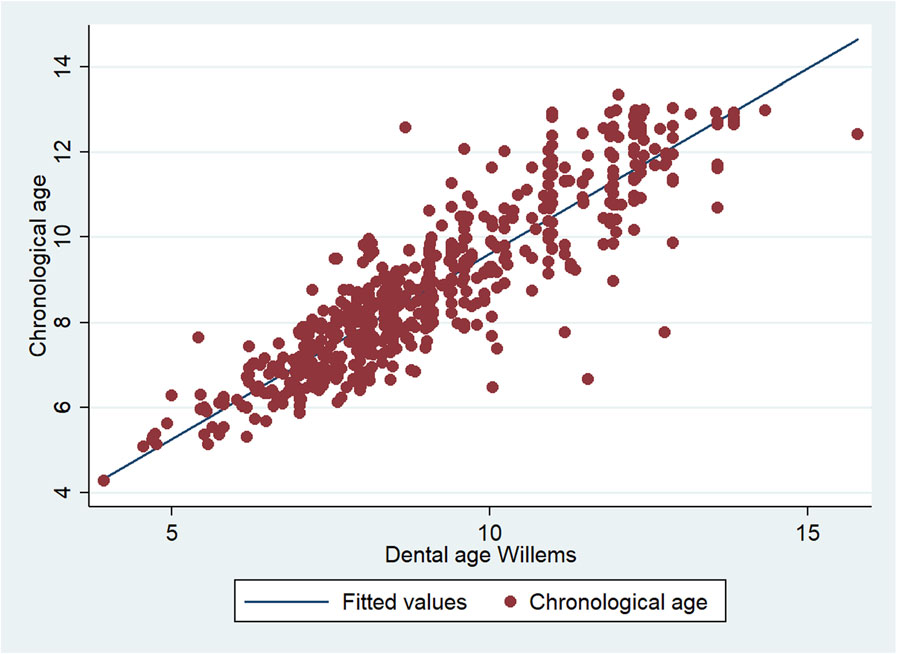
Graphical representation of the Spearman correlation between the dental age of the Willems method and the chronological age

**Fig. 2:**
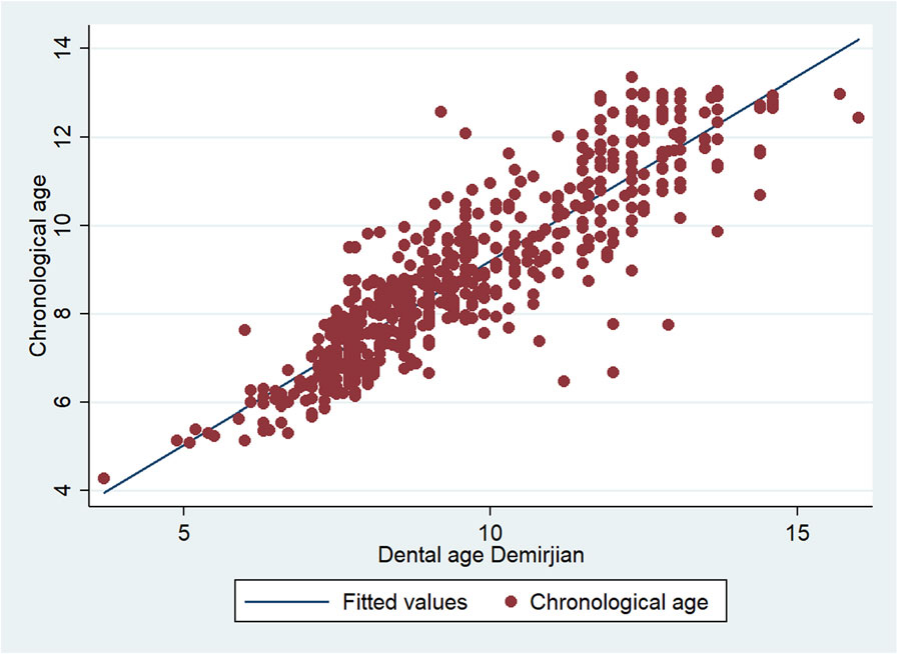
Graphical representation of the Spearman correlation between the dental age of the Demirjian method and the chronological age

**Fig. 3:**
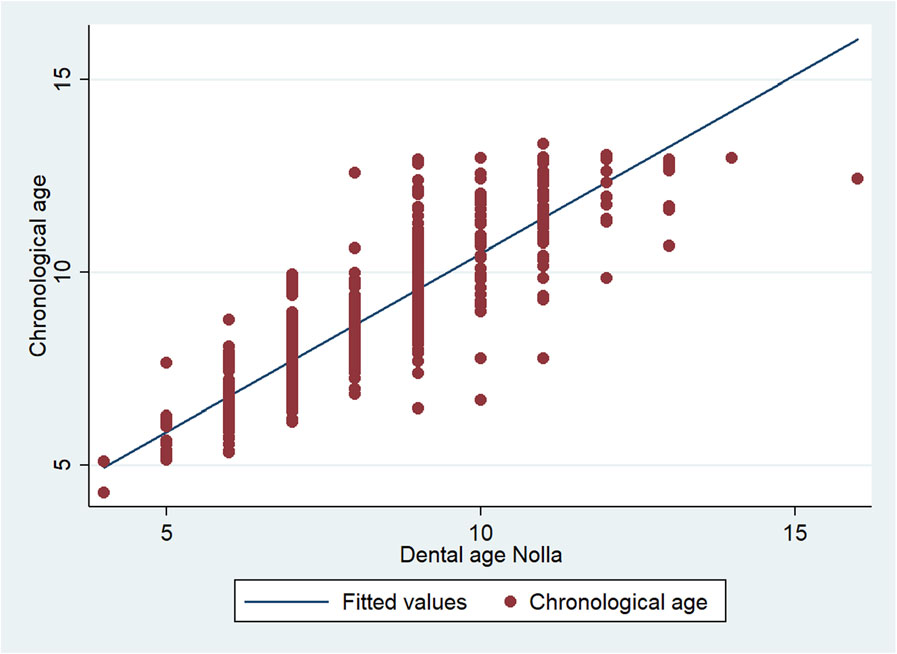
Graphical representation of the Spearman correlation between the dental age of the Nolla method and the chronological age

The graphs show the positive correlation of the calculation of dental age with the three methods with respect to chronological age. The methods in which they are best located at the points near the line, in order of a strong relationship between the variables, are the Willems (Fig. 1), Demirjian (Fig. 2) and Nolla (Fig. 3) methods.

The regression analysis grouped by sex was performed taking into account as a dependent variable the sum of the stages of the seven left mandibular teeth and the conversion to the dental age of each of them to predict the chronological age where it was statistically significant (Table 7).

**Table 7:** Linear regression analysis grouped by sex. Model 1: Demirjian method in girls, Model 2: Demirjian method in boys, Model 3: Willems method in girls, Model 4: Willems method in boys, Model 5: Nolla method in girls and Model 6: Nolla method in boys. * p < 0.001

Model	β	SE β	t	p	β (95% CI)		F	R^2^	R^2^ adjusted	Formula to predict CA
(1) Constant	1.01	0.23	4.33	0.000	0.55	1.47	1133.66*	0.7907	0.7900	CA = 1.01 + 0.81 x DA Demirjian girls
Predictor	0.81	0.02	33.67	0.000	0.77	0.86				
(2) Constant	0.74	0.24	3.09	0.002	0.27	1.22	1191.13*	0.7988	0.7981	CA = 0.74 + 0.85 x DA Demirjian boys
Predictor	0.85	0.02	34.51	0.000	0.80	0.90				
(3) Constant	1.01	0.23	4.43	0.000	0.56	1.47	1183.02*	0.7977	0.7970	CA = 1.01 + 0.87 x DA Willems girls
Predictor	0.87	0.03	34.40	0.000	0.82	0.92				
(4) Constant	0.76	0.24	3.19	0.002	0.29	1.24	1194.54*	0.7993	0.7986	CA = 0.76 + 0.88 x DA Willems boys
Predictor	0 0.88	0.03	34.56	0.000	0.83	0.93				
(5) Constant	1.65	0.25	6.60	0.000	1.16	2.14	844.76*	0.7379	0.7371	CA = 1.65 + 0.90 x DA Nolla girls
Predictor	0.90	0 0.03	29.06	0.000	0.84	0.96				
(6) Constant	0.59	0.25	2.31	0.022	0.09	1.09	1099.80*	0.7857	0.7850	CA = 0.59 + 0.98 x DA Nolla boys
Predictor	0.98	0.03	33.16	0.000	0.92	1.04				

We obtained a predictive capacity of the total variance of the chronological age of the sample of 79.8% girls and 79.9% boys in the scores used for Willems, 79.0% girls and 79.9% boys using Demirjian scores, and 73.8% girls and 78.6% children in the scores used for Nolla.

Table 7 shows the formulas for chronologically forecasting the age using the Demirjian, Willems, and Nolla method scores. Substituting the score obtained with each of the methods in DA we obtain a chronological estimate of age.

## Discussion

In the Spanish population, the Willems method was the most accurate for estimating age. In order of precision, the most appropriate methods for application in boys were the Willems, Nolla and Demirjian methods, and in girls were the Willems, Demirjian and Nolla methods.

### National studies

In Spain, the Demirjian method has been used based on the development of the third molar [18, 29]. However, in the medical-legal environment, the Demirjian method is used based on the stages of the teeth between the left central incisor and the second left molar of the mandible [14]. Its application has been very frequent, and, as in our study, the tendency of the calculation of dental age is towards overestimation [10, 17, 19, 24, 30, 31]. In the study of Melo et al. [19], the precision is very similar to ours, 0.86 and 0.70, respectively. In the case of the study of Feijoo et al. [17, 30], our results obtained greater precision in boys (0.68 versus 0.87) and lower precision in girls (0.70 versus 0.55).

There are two studies in which the Demirjian and Nolla methods have been used together [10, 19]. For both, our results coincide regarding the tendency of the Demirjian method to overestimate, although we obtained greater precision in boys [10], and our results also agreed regarding underestimation with the Nolla method [10, 11, 19].

There is only one study carried out in a population of Spanish origin of 266 children, where the Willems method is applied together with the Demirjian method and the Cameriere method [24]. The results obtained are similar to ours, finding that the Willems and Demirjian methods overestimate and that the Willems method has greater precision. However, our study has a larger sample size, confirming the first results published in the Spanish population.

### International studies

The methods used in this study have been studied worldwide. Most of the findings reported on the calculation of dental age coincide with the trend shown by our results, favoring the external validity of the methods used.

In the case of the Nolla method, we also find underestimation when applied in Brazilians and Croats [32], in Malaysians [33], in Turks [34], in Bangladeshi and British [35] or in Indians [2]. However, there are conflicting results in some of the aforementioned populations, with overestimation being found in the study of Lopes LJ et al. [3] with Brazilians and in the study of Mohammed RB et al. [36] in Indians.

In the case of the Demirjian method, we also find overestimation in Brazilians and Croats [32], in Malaysians [33], in Turks [34], in Bangladeshi and British [35] or in Indians [36].

In the case of the Willems method, we also find overestimation in Bangladeshi and British [35] and in Indians [2]. However, the study carried out by Mohammed RB et al. [36] also in the Indian population reports underestimation with the Willems method.

The application of the Demirjian, Willems and Nolla methods in the same design has been carried out only in three studies [2, 35, 36]. Maber et al. [35] analyzed 946 radiographs of children aged 3 to 16.9 years and Hegde S et al. [2] analyzed1200 radiographs in children between 5 and 15 years. As in our case, the Willems method was the most accurate, and together with the Demirjian method, they overestimated the chronological age. The Nolla method was underestimated in both cases.

In the study by Mohammed RB et al. [36], 760 radiographs were analyzed in children aged 6 to 16 years, and the results showed overestimation by the Demirjian method. However, unlike our findings, overestimation was found with the Nolla method and underestimation with the Willems method.

Studies such as that of Melo et al. [19] analyzed samples of 2641 patients aged between 7 and 21 years. However, the Demirjian method allows the estimation of age only up to 16 years[27]; therefore, the valid sample of this study was 956 children (up to 18 years). In our study, we studied children from 4 to 13 years old, with an equal proportion of boys and girls and a valid sample for the application of the methods used to estimate the dental age.

Dental age could be calculated with the regression models constructed in this study, as on other occasions they have been used in the studies of Diz et al. [1]. In this way, predictions of the chronological age of Spanish children could be made.

### Intra-observer concordance degree

The use of the methods for calculating age through dental maturation shows good or almost perfect degrees of intact-examiner agreement, between 0.79 [10] to 0.94 [4]. Our study obtained degrees of agreement greater than 0.91, so its application demonstrates excellent reliability.

### Limitations

In this study, age ranges with different sample sizes are presented. The study design published by Cortes et al. [11] where the Nolla method has been used independently in the Spanish population has been followed, observing similar results. This decompensation in the sample sizes could mean that in the results of smaller groups, the data should be interpreted with caution. However, in the last age range, with fewer radiographs, the most significant standard deviations are not observed in each of the samples’ age groups.

There are other methods [4, 8, 37, 38] of estimating age based on the growth of the cervical vertebrae, wrist, or finger bones. A positive correlation between bone growth and the state of dental maturation has been shown in numerous studies. However, there is little scientific literature [39] that verifying the correlation of bone growth with the Willems method.

### Legal medical aspects

From a legal medical point of view, it is vitally important to make a favorable estimate of children in the age groups with legal repercussions. In this sense, it is appropriate to use the most accurate methods possible and tend to underestimate it. For this reason, the Willems method is the one that best adapts in this study to the population of Spanish children since, despite tending to overestimate, it is more accurate than the Demirjian and Nolla method.

Given the findings presented here, it would be desirable to use the Willems method in the Spanish population to estimate the dental age.

## Conclusions

The differences in the means between chronological age and dental age are statistically significant in the Willems, Demirjian and Nolla methods; therefore, none of them is completely accurate. In the Spanish population, the use of the Demirjian method for legal and medical purposes is frequent. However, the results of this study reveal that Willems’s method is more related to the actual age, prone to overestimation but still the best of all methods studied.

## Abbreviations

STROBE: Strengthening the Reporting of Observational studies in Epidemiology
JPEG: Joint Photographic Experts Group
CRF: Data collection notebook
CA: Chronological age
DA: Dental age
SD: Standard deviation
Diff.SD: Standard deviation differences
O: Overestimation

## Availability of data and materials

The data analyzed in the current study are available from the corresponding author on reasonable request.
